# Analysis and forecast of hand, foot, and mouth disease epidemic trends in Guangzhou, China, 2013–2023

**DOI:** 10.1371/journal.pone.0333544

**Published:** 2025-09-30

**Authors:** Haipeng Luo, Siyi Zhong, Jiaojiao Liu, Hui Wang, Zhoubin Zhang

**Affiliations:** 1 School of Public Health, Sun Yat-sen University, Guangzhou City, Guangdong Province, China; 2 Department of Communicable Disease Control and Prevention, Guangzhou Center for Disease Control and Prevention (Guangzhou Health Supervision Institute), Guangzhou, China; 3 School of Public Health, Southern Medical University, Guangzhou City, Guangdong Province, China; Shanxi University, CHINA

## Abstract

Hand, foot, and mouth disease (HFMD) primarily affects children under the age of five and has emerged as a significant public health concern in China. This study examines HFMD incidence trends across various gender and age demographics in Guangzhou from 2013 to 2023, providing a comprehensive overview to inform future prevention and management strategies. Utilizing epidemiological data from the National Notifiable Infectious Disease Reporting Information System, we employed Joinpoint regression modeling to assess variations in incidence rates among different demographic cohorts and developed a Seasonal Autoregressive Integrated Moving Average (SARIMA) model to analyze case trends and project future occurrences. The findings revealed biennial peaks and notable seasonality, indicating cases across all age groups, though predominantly among children under five. Joinpoint regression analysis demonstrated a significant decrease in incidence rates for children younger than one year (Average Annual Percent Change [AAPC]=−13.50%, *P* < 0.001) and those aged one year (AAPC = −10.35%, *P* = 0.002), while incidence remained stable for children aged two to five years. Conversely, a significant upward trend was identified in children aged six years and older (AAPC = 13.09%, *P* = 0.019). Gender-specific trends closely resembled these overall patterns, with both males and females experiencing similar percentage changes in incidence rates. The SARIMA model indicates a projected decline in HFMD cases for 2024, followed by a phase of relative stability characterized by minor fluctuations. These results offer crucial insights into HFMD trends, illustrating the effectiveness of current preventive measures in reducing incidence among younger children while underscoring the necessity for targeted interventions to address the rising trends in older age groups. By leveraging these findings, health authorities can refine control strategies and prioritize public health responses to effectively mitigate HFMD outbreaks.

## Introduction

Hand, foot, and mouth disease (HFMD) is an infectious condition caused by human enteroviruses and coxsackieviruses, predominantly impacting children under the age of five [1,2]. Clinical presentation includes fever, vesicular eruptions on the hands, feet, and buttocks, as well as ulcers in the oral mucosa [3]. Since its initial identification in New Zealand in 1957, HFMD has proliferated and is now endemic to the Asia-Pacific region, encompassing countries such as China, Japan, and Malaysia [4–8]. In China, HFMD poses a considerable public health challenge [9]. Following its designation as a Category C notifiable infectious disease in 2008, HFMD has consistently ranked among the leading reportable diseases in terms of morbidity and mortality. Guangzhou, situated in the Pearl River Delta region of southern China, reports one of the highest incidence rates of HFMD in the nation.

A comprehensive understanding of disease trends is essential for effectively responding to future changes in HFMD patterns. Previous investigations have indicated a declining trend in HFMD incidence and a shift towards later age of onset [10,11]. However, there is a scarcity of studies that have thoroughly examined the trends in HFMD incidence across various gender and age demographics.

This study aimed to explore the trends in HFMD incidence among different gender and age cohorts in Guangzhou from 2013 to 2023. We utilized Joinpoint regression modeling to assess changes in incidence rates among these groups. Additionally, we employed a SARIMA model to analyze case trends and project the incidence rate for subsequent two years. Our findings will support health authorities in refining HFMD prevention and control strategies, enabling them to adjust their public health priorities for a more effective response to the epidemic.

## Methods

### Study area

Guangzhou, situated at coordinates 22°26′-23°56′N and 112°57′-114°3′E in southern China, lies at the northern periphery of the Pearl River Delta, adjacent to the South China Sea. As the capital of Guangdong Province, it serves as a pivotal center for trade, commerce, and transportation in South China. Renowned for its rich cultural diversity, Guangzhou attracts a significant number of immigrants from both domestic and international backgrounds. This substantial influx of population results in a pronounced occurrence of infectious diseases, notably including one of the highest rates of HFMD in China.

### Data collection

Data regarding cases of HFMD, including fundamental demographic information (sex, date of birth), disease severity (classified as mild or severe), and key dates (symptom onset and diagnosis where applicable), were extracted from the National Notifiable Infectious Disease Reporting Information System for the period 01/01/2013 to 31/12/2023. These data were accessed for research purposes on 20/08/2024. No personally identifiable information was collected or retained during or after the study. Annual demographic statistics stratified by gender and age group for the same period were sourced from the *Guangzhou Statistical Yearbook* [12].

### Statistical methods

#### Joinpoint regression.

The Joinpoint regression model is employed for trend analysis in time series data or other ordered data, and it finds extensive application in epidemiological trend studies [13]. This model was first introduced by Kim in 1998, and its core concept involves establishing piecewise regression based on the temporal characteristics of disease distribution. It segments the study period into different intervals through several connecting points and performs trend fitting and optimization for each interval, thus providing a more detailed assessment of specific disease variation characteristics across the overall time frame [14]. This study utilizes the Joinpoint regression model to analyze the changes in the reporting incidence of hand, foot, and mouth disease in Guangzhou from 2013 to 2023. The model with the smallest WBIC value was determined to be the optimal model. Detailed model selection information can be found in in S1 File.

By calculating the annual percent change (APC), average annual percent change (AAPC), and their 95% confidence intervals (*CI*), we describe the variation trends in incidence rates by gender and age groups, assessing statistical significance where *P *< 0.05 indicates meaningful differences. APC serves to describe the trend in incidence rate variations over a specific time frame, while AAPC represents the overall average change in incidence rates during the study period. When the number of turning points is zero, APC equals AAPC. An APC or AAPC greater than zero indicates an upward trend in incidence rates during that period; conversely, if APC or AAPC is less than zero, it signifies a downward trend; when APC or AAPC equals zero, it indicates stability in incidence rates during that timeframe [15].

#### SARIMA model.

The Autoregressive Integrated Moving Average (ARIMA) model, established in the early 1970s by George Box in the United States and Gwilym Jenkins in the United Kingdom, is a prominent tool for predicting infectious diseases [16–18]. It encompasses three primary components: the Autoregressive (AR) model, the Moving Average (MA) model, and the Autoregressive Moving Average (ARMA) model. In this study, we initially constructed a seasonal ARIMA model [ARIMA (p,d,q) (P,D,Q)s], whereby (p), (d), and (q) denote the orders of the autoregressive, integrated, and moving average elements of the model, respectively. (P) signifies the count of seasonal autoregressive terms, (D) indicates the number of seasonal differences, (Q) defines the quantity of seasonal moving average components, and (S) represents the seasonal period.

The model was developed using R software (version 4.4.0) with the packages “tseries,” “forecast,” “ggplot2,” and “Metrics.” The optimal model was selected using the “auto.arima” function, where a two-tailed *P* < 0.05 was regarded as statistically significant. Additionally, we employed the Ljung-Box test to evaluate whether the residuals of the model are white noise and inspected the ACF and PACF of the residuals to further assess the stochastic, smoothed, and seasonal influences on the time series data. Furthermore, we retained data from week 27 of 2023 onward as a validation set, utilizing absolute percentage error (mean absolute percentage error, MAPE) and root mean square error (RMSE) for assessing the forecasting performance. We also projected the incidence of hand, foot, and mouth disease over the subsequent two years.

## Results

### Epidemiological overview of hand, foot, and mouth disease

**Fig 1:**
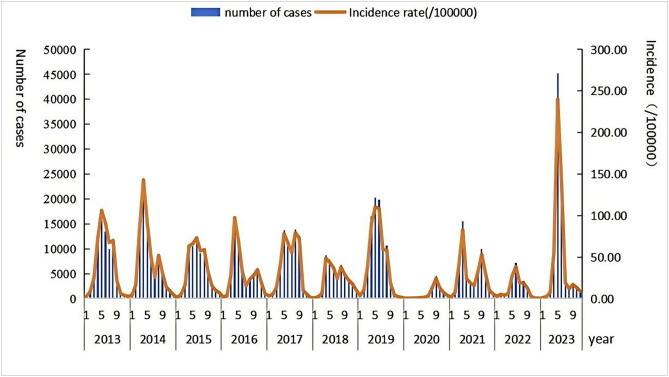
Temporal distribution of HFMD incidence in Guangzhou from 2013 to 2023. The figure illustrates the monthly number of cases (blue bars) and the corresponding incidence rate per 100,000 population (orange line) from 2013 to 2023. The x-axis is marked by years and key monthly intervals (e.g., “1 5 9” denotes the 1st, 5th, and 9th months within each year), depicting the temporal trends and cyclical fluctuations in disease occurrence over the decade.

Between 2013 and 2023, Guangzhou documented a cumulative total of 694,145 cases of HFMD, resulting in an average annual incidence rate of 403.60 per 100,000 individuals. Among these cases 20 were categorized as severe. The incidence of HFMD demonstrated biennial peaks and pronounced seasonality. With the exception of 2020, each year exhibited a bimodal distribution. Cases were recorded across all age demographics, with children under the age of five representing 92.35% of the overall cases (Fig 1).

### Analysis of trends in HFMD incidence rates

#### Gender-based trends in incidence rates.

**Fig 2:**
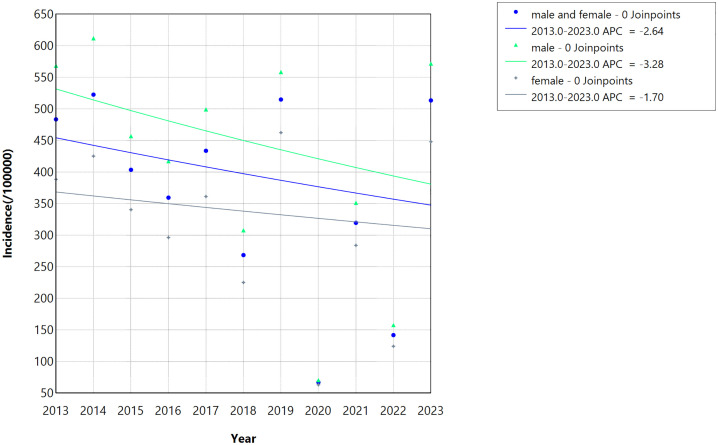
Gender-specific incidence trends of HFMD in Guangzhou from 2013 to 2023. The figure depicts the temporal trends in disease incidence (per 100,000 population) between 2013 and 2023, analyzed using Joinpoint regression and stratified by gender (male, female) as well as the overall population. The blue dots and their connecting line represent the overall population; green triangles and their connecting line denote males; and black dots and their connecting line signify females. All groups exhibit “0 Joinpoints,” indicating a single linear trend over the period. Furthermore, this visualization captures the year-on-year changes in the data between males and females during this period.

During the study period (2013–2023), the reported incidence rate of HFMD in Guangzhou remained stable, with an AAPC of −2.64% that lacked statistical significance (*P* > 0.05) (Fig 2). When stratified by gender, the incidence rates among males and females mirrored this stable trend, with no statistically significant differences observed (*P* > 0.05).

#### Trends in incidence rates by age groups.

The analysis indicates that the incidence rates of HFMD in Guangzhou display notable trends when evaluated across different age categories. As illustrated in Table 1, the incidence rates for the groups “under 1 year old” and “1 year old” reveal a consistent decline, with AAPC of −13.50% and −10.35%, respectively, both of which are statistically significant (*P* < 0.05). Conversely, the incidence rates for the “2 years old,” “3 years old,” “4 years old,” and “5 years old” groups remained stable, reflecting no statistically significant variations (*P* > 0.05). In contrast, the “6 years old or older” group demonstrated a general upward trend, with an AAPC of 13.09%, which is also statistically significant (*P* < 0.05).

**Table 1:** Age-specific incidence trends of HFMD in Guangzhou from 2013 to 2023.

Age group	AAPC(95%*CI*)	*P* value
Under 1 year old	−13.50%(−21.52% to −6.42%)	<0.001
1 year old	−10.35%(−17.97% to −3.66%)	0.002
2 years old	−7.56%(−18.12% to 2.48%)	0.133
3 years old	−6.44%(−15.67% to 3.45%)	0.170
4 years old	−0.20%(−10.27% to 12.74%)	0.883
5 years old	−0.92%(−8.95% to 10.16%)	0.951
6 years old or older	13.09%(2.49% to 30.10%)	0.019

AAPC, average annual percent change; 95%*CI*, 95% confidence intervals.

#### Trends in incidence rates by gender and age groups.

As presented in Table 2, the trends in incidence rates across various age groups from 2013 to 2023, when analyzed by gender, closely mirrored the overall trends observed. In the “under 1 year old” and “1 year old” categories, both male and female incidence rates of HFMD demonstrated a downward trajectory, with AAPC of −14.12% and −12.54% for the “under 1 year old” group, and −11.04% and −9.35% for the “1 year old” group, respectively. These trends were statistically significant (*P* < 0.05). In contrast, the incidence rates for the “2 years old,” “3 years old,” “4 years old,” and “5 years old” categories remained relatively stable, showing no statistically significant changes (*P* > 0.05). Conversely, in the “6 years old or older” group, both male and female incidence rates exhibited an upward trend, with AAPC of 13.23% and 12.85%, respectively, and these increases were statistically significant (*P* < 0.05).

**Table 2:** Age-specific and gender-specific incidence trends of HFMD in Guangzhou, 2013–2023.

Age group	Male		Female	
	AAPC(95%*CI*)	*P* value	AAPC(95%*CI*)	*P* value
Under 1 year old	−14.12%(−22.11% to −7.16%)	<0.001	−12.54%(−20.53% to −5.15%)	0.001
1 year old	−11.04%(−18.40% to −4.69%)	<0.001	−9.35%(−17.11% to −2.37%)	0.008
2 years old	−7.86%(−18.76% to 2.02%)	0.119	−7.01%(−16.95% to 3.00%)	0.156
3 years old	−6.56%(−15.08% to 2.37%)	0.136	−6.09%(−15.25% to 4.11%)	0.209
4 years old	−0.63%(−10.98% to 12.19%)	0.970	0.62%(−9.03% to 13.71%)	0.740
5 years old	−0.53%(−8.38% to 10.28%)	0.908	−1.22%(−8.92% to 9.84%)	0.989
6 years old or older	13.23%(1.52% to 31.59%)	0.036	12.85%(1.69% to 30.49%)	0.030

AAPC, average annual percent change; 95%*CI*, 95% confidence intervals.

### Trends and predictions of HFMD using the SARIMA model

A SARIMA model was developed, resulting in a SARIMA (2,0,2) (0,0,1) configuration. The residuals were assessed using the Ljung-Box test, while the ACF and PACF plots indicated a statistic of 90.098 alongside a *P*-value of 0.7731, both exceeding the significance threshold. This suggests that the residual series adequately represents white noise (see Figs S1 and S2 in S2 File). The Augmented Dickey-Fuller Test returned a Dickey-Fuller value of −4.4257 with a *P*-value below 0.01, confirming that the time series is stationary. Furthermore, all model parameters were found to be statistically significant (*P* < 0.05) (Table S1 in S2 File).

**Fig 3:**
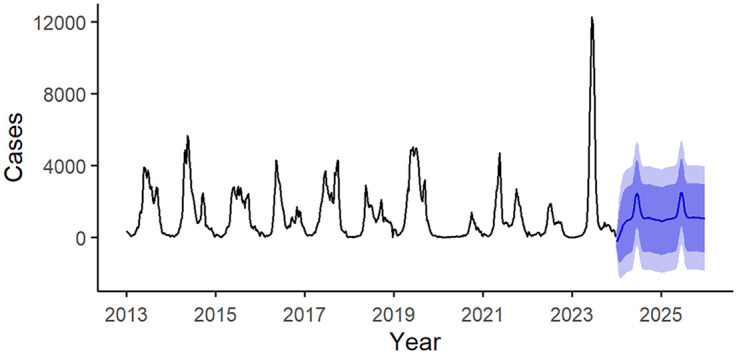
Temporal Trends and Forecasting of Hand, Foot, and Mouth Disease. This figure depicts the spatiotemporal dynamics of disease cases from 2013 to 2025. The black curve represents the actual historical case counts over the period 2013–2023. The blue curve and its surrounding shaded area correspond, respectively, to the projected case counts for 2024–2025 and their associated confidence intervals.

Subsequently, we employed the optimal model to project HFMD cases for the ensuing two years, with results illustrated in Fig 3. The findings indicate a downward trend in HFMD cases for 2024, followed by a phase of relative stability characterized by minor fluctuations.

## Discussion

This study employed the Joinpoint regression model and the SARIMA model to analyze the epidemic trends of HFMD in Guangzhou from 2013 to 2023, and to forecast the outbreak trajectories over the subsequent two years. The findings indicate that while the overall incidence rate remained relatively stable during the research period, notable disparities in incidence trends were observed across different age groups.

Epidemiological trend analysis of HFMD in Guangzhou over the 11-year period from 2013 to 2023 demonstrates a non-significant downward tendency in the average annual percentage change of incidence. This trend exhibits consistency with the epidemiological pattern observed in Jiangsu Province, China during 2009–2023, while showing divergence from that recorded in Shanghai between 2011–2021 [19,20], thereby suggesting that the time frame of 2021–2023 may represent a critical inflection point in its declining trajectory. Such variations in incidence trends are potentially linked to alterations in the pathogen profile of HFMD-causing viruses, with particular relevance to the alternating prevalence of distinct enterovirus serotypes within the population [11]. Notably, the substantial reduction in HFMD incidence in 2020 is likely attributable to the implementation of effective non-pharmaceutical interventions during the COVID-19 pandemic, including social distancing protocols, widespread mask-wearing, and temporary school closures, which collectively suppressed HFMD transmission [21]. However, a gradual resurgence in HFMD incidence was observed from 2021 to 2023, with a marked peak recorded in 2023. This rebound may be influenced by the post-pandemic recovery of social interactions and fluctuations in children's immune status [22,23]. Given the significant upsurge in HFMD cases in 2023 compared to preceding years, it is plausible that this shift is closely associated with adjustments in pandemic management strategies and public health intervention policies [24,25]. Temporal variations in peak incidence were also evident: the peak period occurred between weeks 22 and 43 in 2022, whereas in 2023, it advanced to weeks 18–32. This discrepancy may stem from multiple factors, including the revitalization of social activities post-pandemic, increased frequency of collective gatherings among children, and the gradual normalization of case reporting mechanisms [26,27]. As pandemic control measures were relaxed, the resumption of regular operations in educational institutions and childcare facilities expanded opportunities for virus transmission, thereby contributing to the rise in reported cases. Additionally, the case reporting system, which had been partially suppressed during the pandemic, progressively returned to standard practice in 2023, potentially facilitating more timely and comprehensive recording of cases and resulting in more concentrated data distribution compared to previous years [28].

This study further investigates HFMD incidence trends across different gender groups. Results from Joinpoint regression analysis demonstrate that the AAPC estimates of incidence rates for both males and females yield no statistically significant differences. This suggests that prior interventions have a minimal impact on the gender differences observed. Current interventions primarily encompass population-wide strategies, including health education, vaccination initiatives, and non-pharmaceutical measures such as hand hygiene practices. Existing research indicates no significant gender-based disparities in adherence to these intervention measures [29,24]. However, consistent with previous nationwide studies, this study reveals that male HFMD incidence consistently exceeds that of females [30]. This gender-related pattern may be associated with behavioral characteristics in boys, such as more frequent participation in outdoor activities and less rigorous adherence to hygiene practices, as well as biological factors including variations in immune response profiles [31,32]. Additionally, emerging evidence suggests that male patients exhibit relatively higher transmissibility compared to female patients [32]. These collective findings underscore the necessity of implementing more gender-tailored intervention strategies for HFMD control. Specifically, there is a need to strengthen targeted health education for male individuals, enhance compliance with hygiene practices, and optimize vaccination strategies within high-risk populations to effectively mitigate HFMD transmission.

Furthermore, this study elucidates the long-term incidence trends of HFMD across different age groups. The findings reveal a significant decline in HFMD incidence among children aged 1 year and younger, as well as those in the 1-year-old age group. Conversely, a significant upward trend has been observed in the incidence among children aged 6 years and older. This age-specific pattern is consistent with previous nationwide studies in China, which have indicated an increasing prevalence of HFMD among populations aged 3 years and older [11]. The substantial decrease in HFMD incidence among children aged 1 year and younger likely reflects the effectiveness of public health interventions. For instance, since 2016, China has implemented an EV-A71 vaccination strategy targeting young children, which has not only reduced the number of severe cases but may also have curtailed viral transmission [33]. Additionally, this age group typically has a limited range of activities, benefiting from greater familial protection. Moreover, institutions such as kindergartens enforce stringent HFMD preventive measures, which may further contribute to the decline in incidence [34,35]. Previous studies have also highlighted that non-pharmaceutical interventions, including hand hygiene practices and environmental disinfection, are particularly effective in controlling HFMD transmission among children under 2–3 years of age [36]. In contrast to the decreasing trend observed in younger age groups, HFMD incidence among children aged 6 years and older has gradually increased. This upward trend may be linked to heightened exposure risks associated with the rising incidence of HFMD among adults [37,38]. Furthermore, the recent shift in the pathogenic spectrum—from an EV-A71-dominant pattern to a predominance of CVA6 and CVA10—may result in milder clinical manifestations in some cases. This could lead to reduced healthcare-seeking behavior among infected children, thereby influencing data accuracy. Conversely, children aged 6 years and older, who engage in a broader range of activities, may have benefited from enhanced screening opportunities during the pandemic. Coupled with improved diagnostic capabilities, this may have contributed to an increase in HFMD detection rates [39–42]. While the specific mechanisms underlying this increase require further investigation, it underscores the necessity of strengthening awareness of HFMD preventive measures among older children to prevent wider transmission across age groups. Additionally, the study finds no significant change in HFMD incidence among children aged 2–5 years. This suggests that control measures targeting this age bracket may have achieved a certain degree of effectiveness; however, continuous monitoring and sustained preventive efforts remain essential to prevent disease resurgence.

In addition to analyzing historical incidence trends, this study employed SARIMA models to forecast future HFMD dynamics. The forecasting results indicated that after reaching a peak in 2023, the number of HFMD cases is expected to show a declining trend, which aligns with the historical pattern of HFMD experiencing periodic peaks approximately every other year. Nevertheless, the projected case numbers remain at relatively elevated levels, emphasizing the importance of sustained monitoring and preventive measures to prevent the occurrence of large-scale outbreaks in the future. Based on the comprehensive findings of this study, several targeted recommendations are proposed. Firstly, enhanced surveillance of HFMD cases should be implemented for children aged six years and older, with particular focus on school environments and extracurricular activity settings, accompanied by the enforcement of more stringent preventive protocols. Secondly, there is an urgent need to optimize vaccine strategies, including accelerating the research, development, and promotion of vaccines targeting CVA6 and CVA10, to effectively reduce the transmission of HFMD caused by non-EV71 enteroviruses. Additionally, public health intervention measures should be further strengthened, with specific emphasis on promoting good personal hygiene practices among preschool and school-aged populations. This includes advocating for regular handwashing, improving environmental cleanliness standards, and minimizing close contact in high-risk groups to mitigate the risk of viral transmission.

This study has certain limitations. Firstly, the HFMD cases analyzed may only represent a fraction of the actual infections and cases within the population. Due to the recent decrease in severe cases, some patients with mild symptoms may not seek medical treatment, resulting in an apparent decline in HFMD trends. Nevertheless, HFMD case data were extracted from China’s National Notifiable Infectious Disease Reporting Information System, a comprehensive and authoritative surveillance platform. We made strenuous efforts to maximize the inclusion of all reported cases during the study period, thereby enhancing the reliability of our results. While we acknowledge the potential underreporting of mild cases, this national surveillance system still provides a robust foundation for capturing the overall epidemiological trends of HFMD. Secondly, given that HFMD cases predominantly occur among children, the reported decline in case numbers may also correlate with the decreasing birth rate in Guangzhou, which may not accurately reflect the actual temporal trends. However, Joinpoint analysis employed age-stratified incidence rates, with age-specific population data used as denominators for each group. This methodological approach effectively accounted for the impact of birth rate fluctuations on the structure of the susceptible population, thereby minimizing interference from underlying population changes.

## Conclusions

From 2013 to 2023, the incidence rate of HFMD in Guangzhou has shown a consistent trend, primarily impacting children under five years of age. Nevertheless, this overall consistency obscures notable variations among different age demographics; specifically, the incidence rates for infants under one year and children aged one have demonstrated a downward trajectory, while there has been an increase in rates for children aged six years and older. Predictions suggest that HFMD cases may decrease and stabilize over the next two years. It is essential to continually enhance surveillance efforts and implement comprehensive public health interventions to facilitate the timely identification of shifts in disease trends. This approach will aid health authorities in refining control strategies and aligning public health priorities, thus ensuring more effective responses to HFMD outbreaks.

## Supporting information

S1 File Selection of Joinpoint Regression Models Using the Weighted Bayesian Information Criterion (WBIC). (DOCX)

S2 File Selection and validation of ARIMA parameters. (DOCX)
