# Supplementary material for: Analysis and forecast of hand, foot, and mouth disease epidemic trends in Guangzhou, China, 2013–2023
Source: PLoS One. 2025 Sep 30;20(9):e0333544. doi: 10.1371/journal.pone.0333544 (PMC12483251; doi:10.1371/journal.pone.0333544)

As of version 5.0, the Weighted BIC is the default for use in the Joinpoint software. The model with the smallest WBIC value was determined to be the optimal model. WBlC is calculated as ((1 - Weight) * BlC) + (BlC3 * Weight). Figure S1 shows the trend in incidence rates across the entire population; Figures S2–S3 show the trends in incidence rates by gender; Figures S4–S10 show the trends in incidence rates by age group; Figures S11–S24 show the trends in incidence rates by gender and age group. The specific details are as follows:

Figure S1. Trends in incidence rates across the entire population
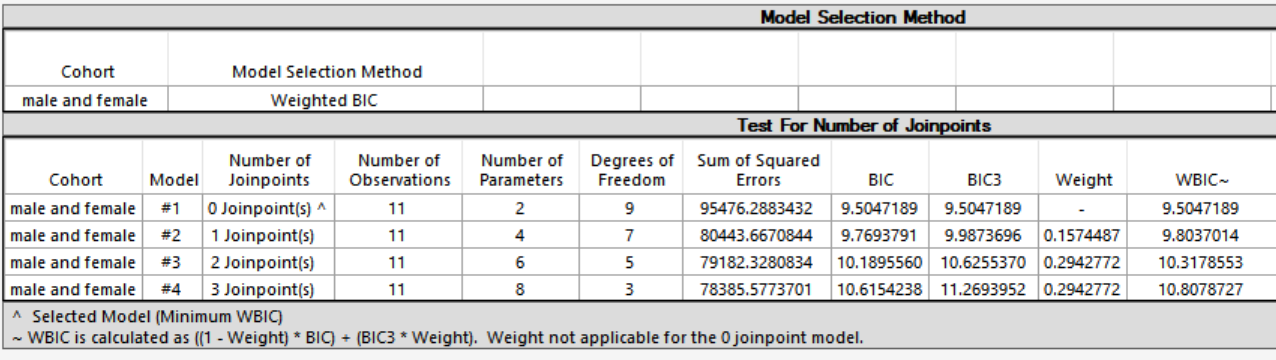


Figure S2. Trends in male incidence rates
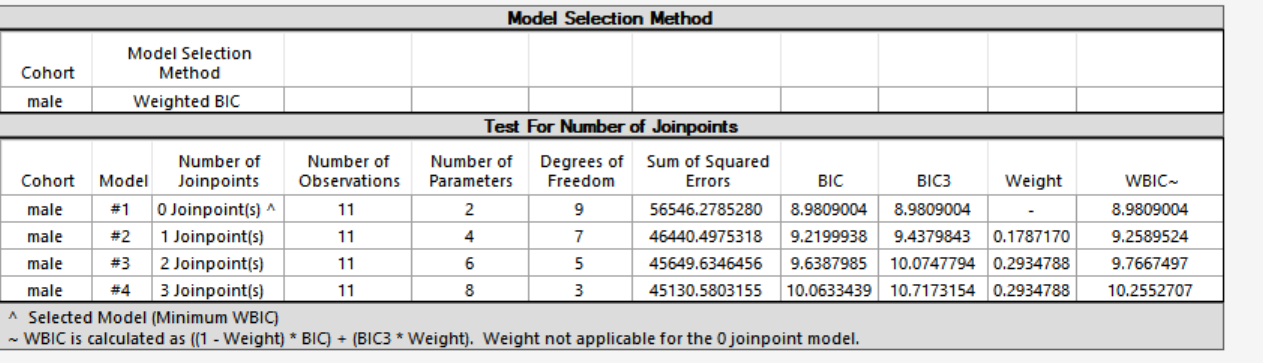


Figure S3. Trends in female incidence rates
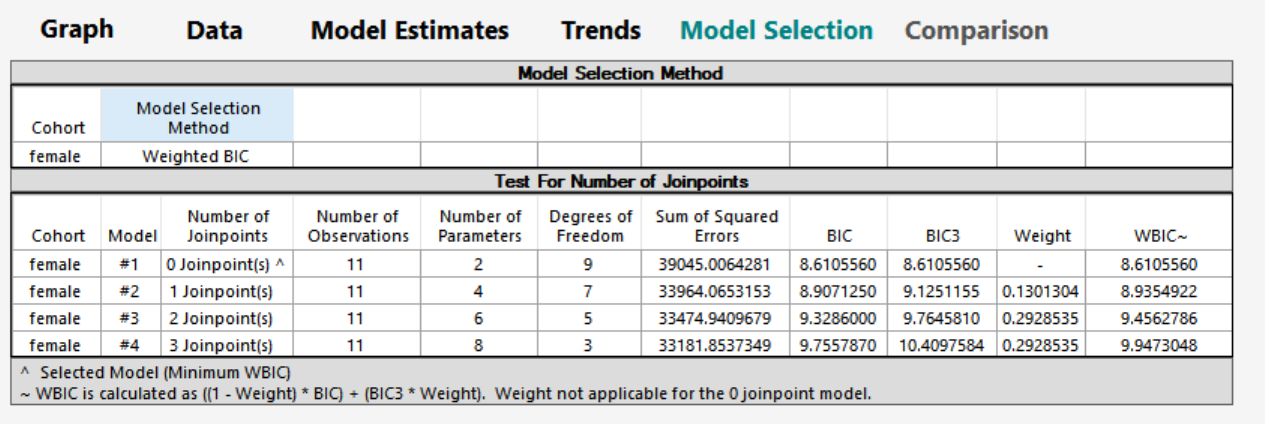


Figure S4. Trends in incidence rates for less than 1 year old


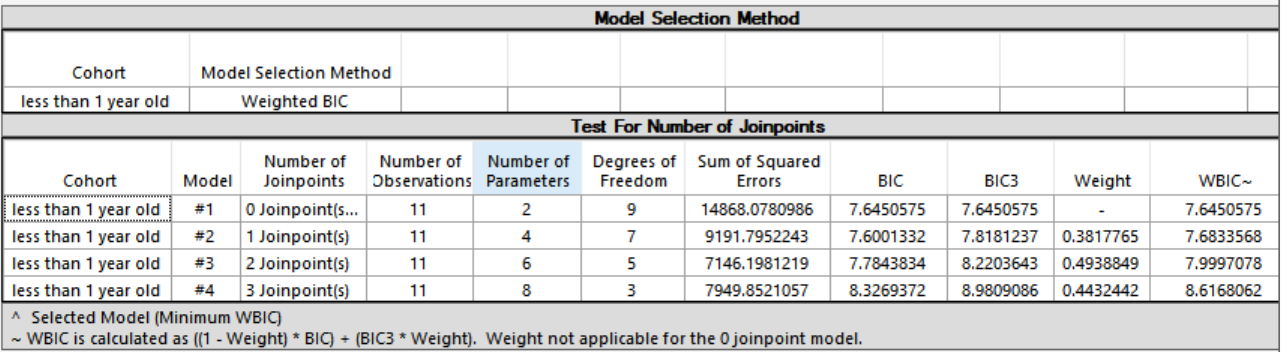


Figure S5. Trends in incidence rates for 1 year old


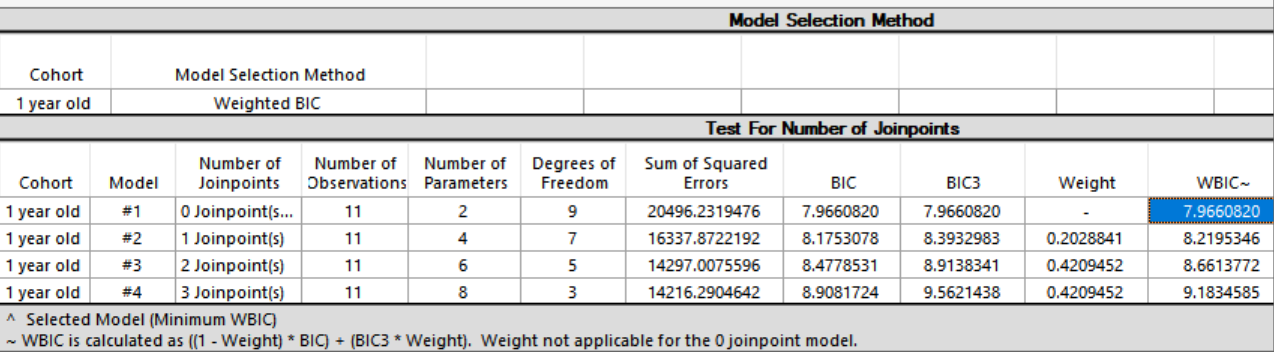


Figure S6. Trends in incidence rates for 2 years old


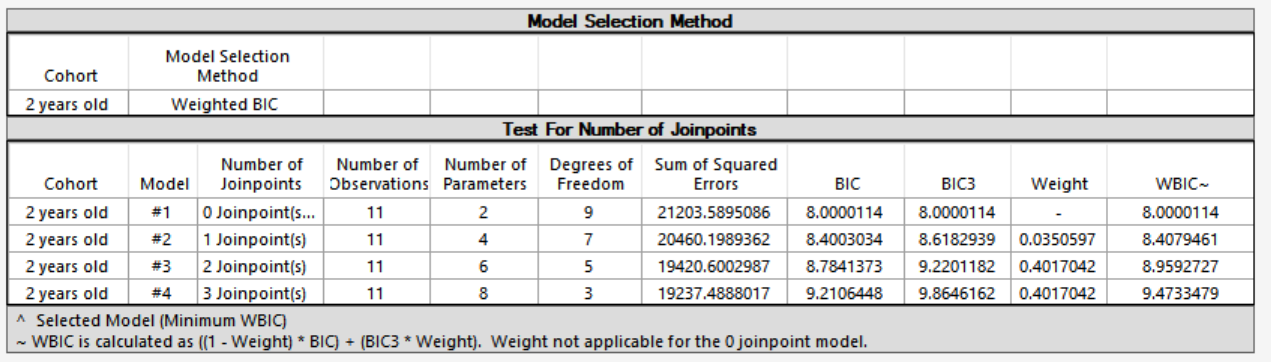


Figure S7. Trends in incidence rates for 3 years old


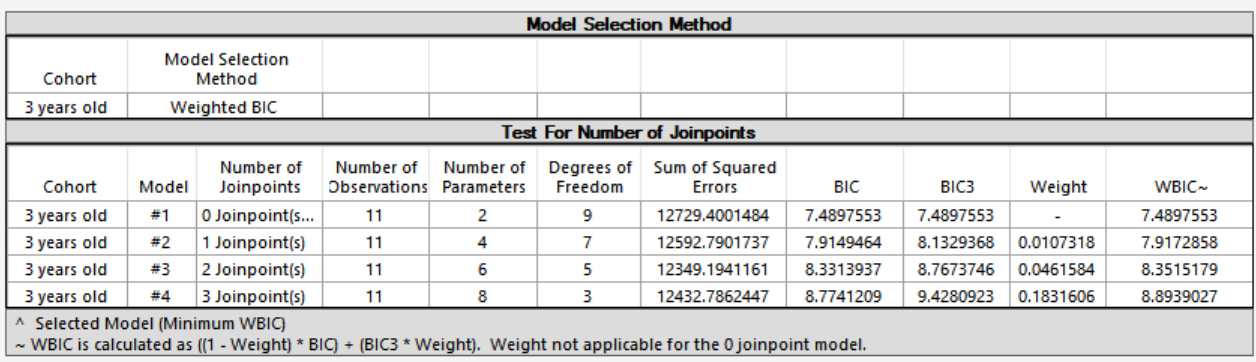


Figure S8. Trends in incidence rates for 4 years old


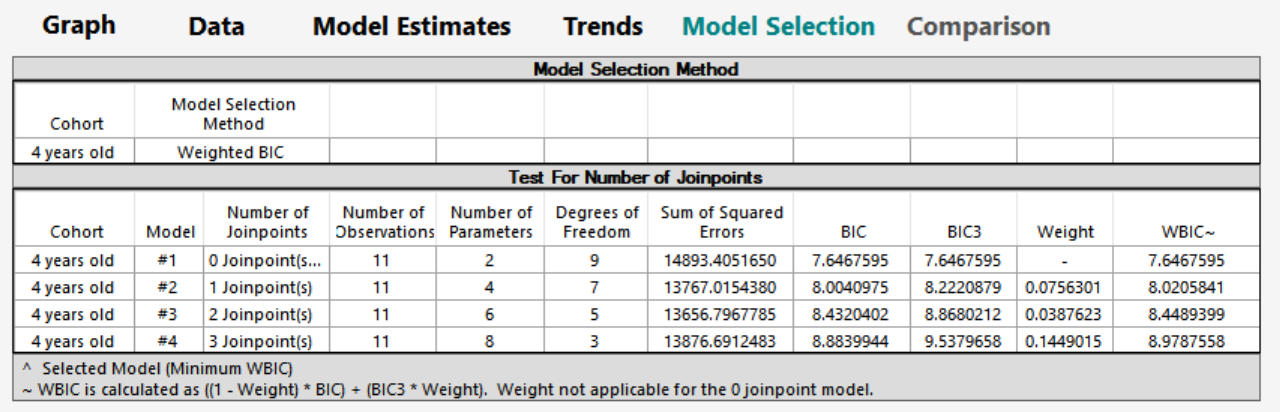


Figure S9. Trends in incidence rates for 5 years old


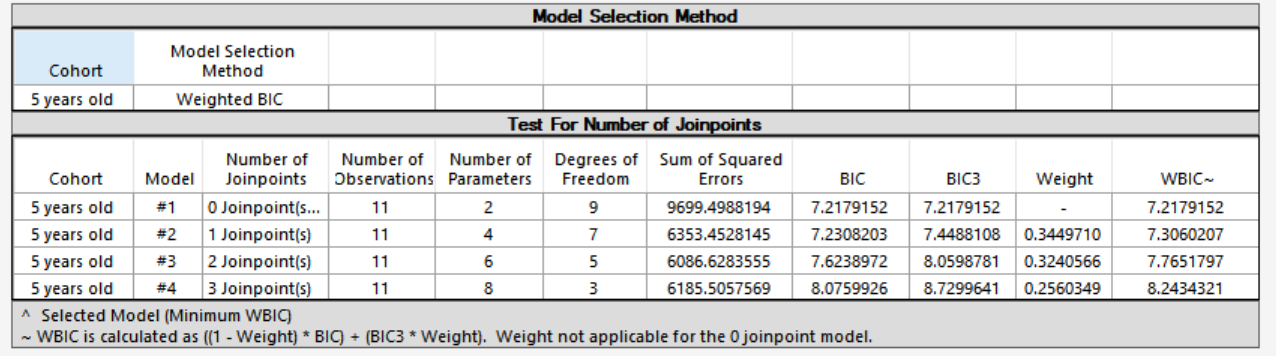


Figure S10. Trends in incidence rates for 6 years old or older


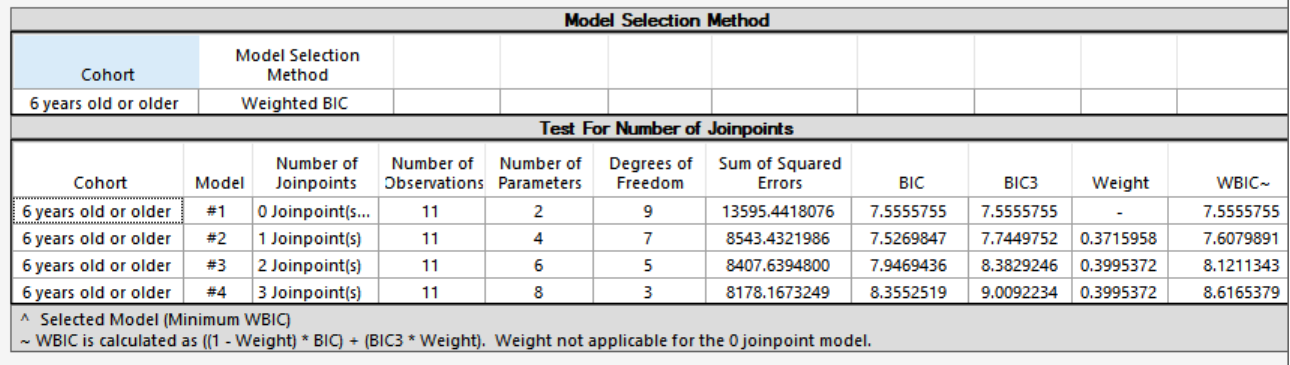


Figure S11. Trends in incidence rates among male aged less than 1 year old
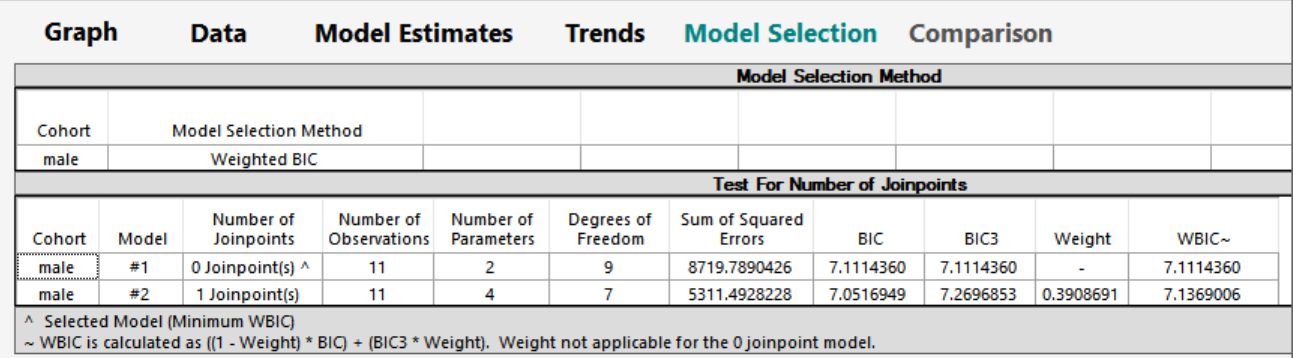


Figure S12. Trends in incidence rates among female aged less than 1 year old
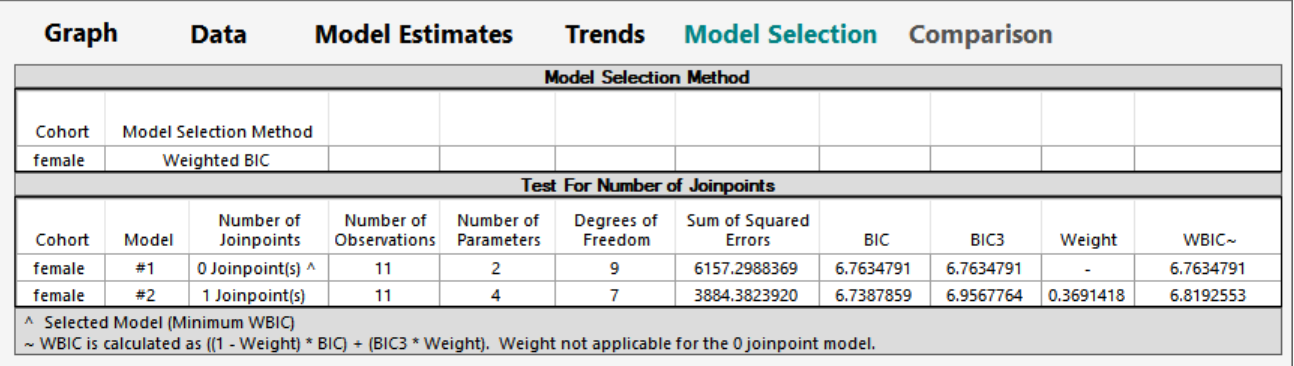


Figure S13. Trends in incidence rates among male aged 1 year old
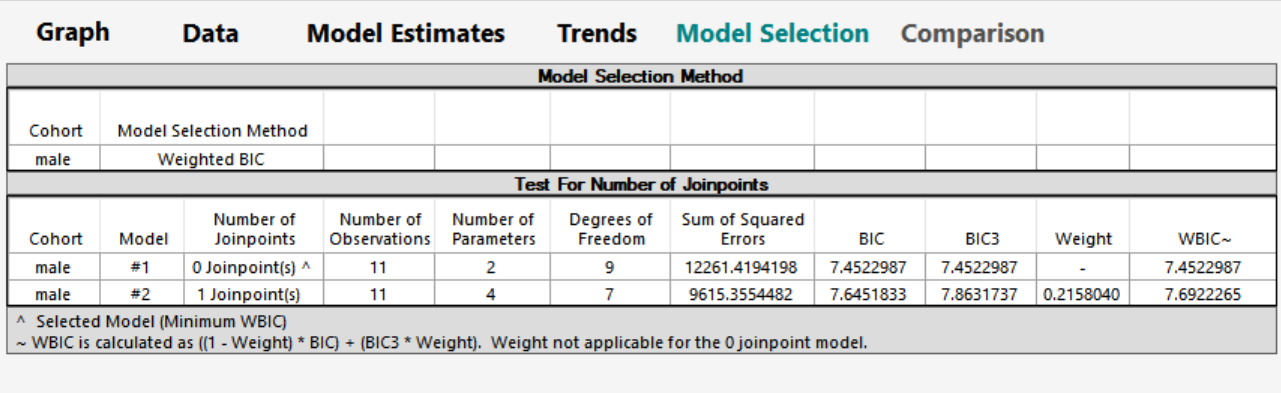


Figure S14. Trends in incidence rates among female aged 1 year old
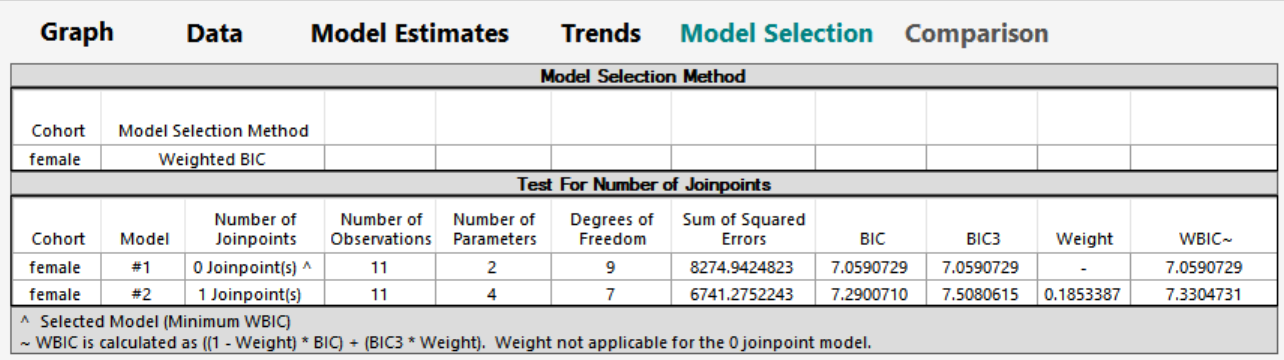


Figure S15. Trends in incidence rates among male aged 2 years old
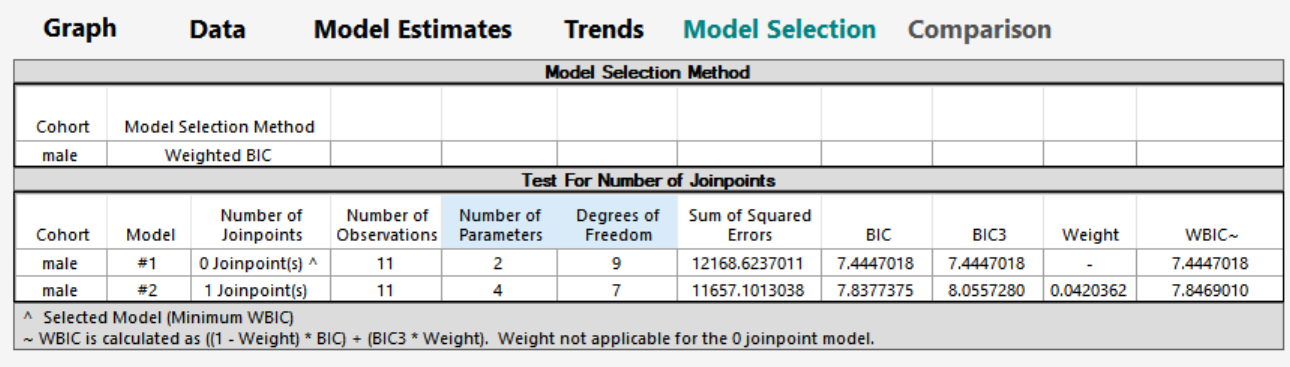


Figure S16. Trends in incidence rates among female aged 2 years old
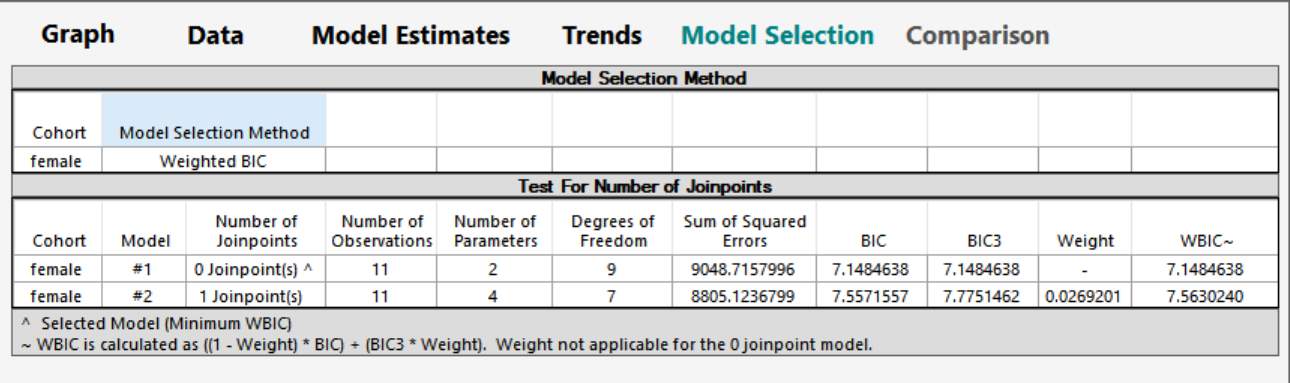


Figure S17. Trends in incidence rates among male aged 3 years old
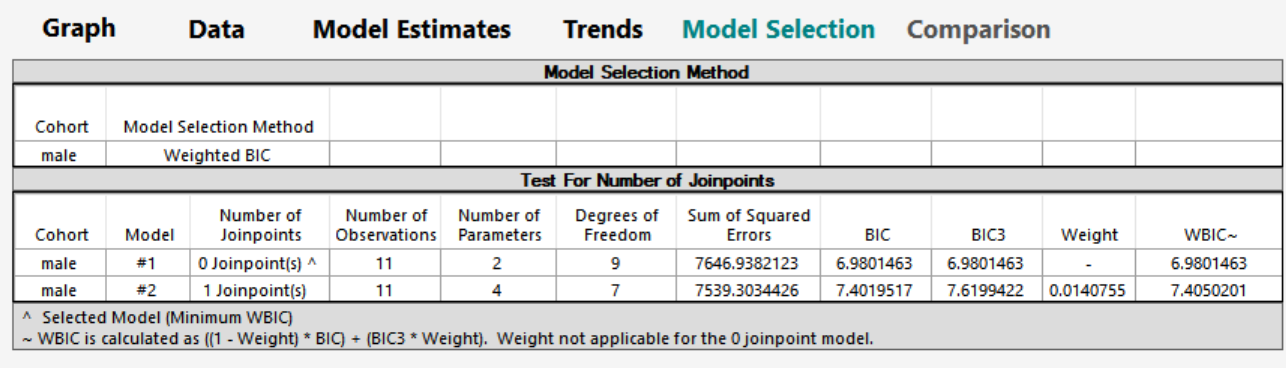


Figure S18. Trends in incidence rates among female aged 3 years old
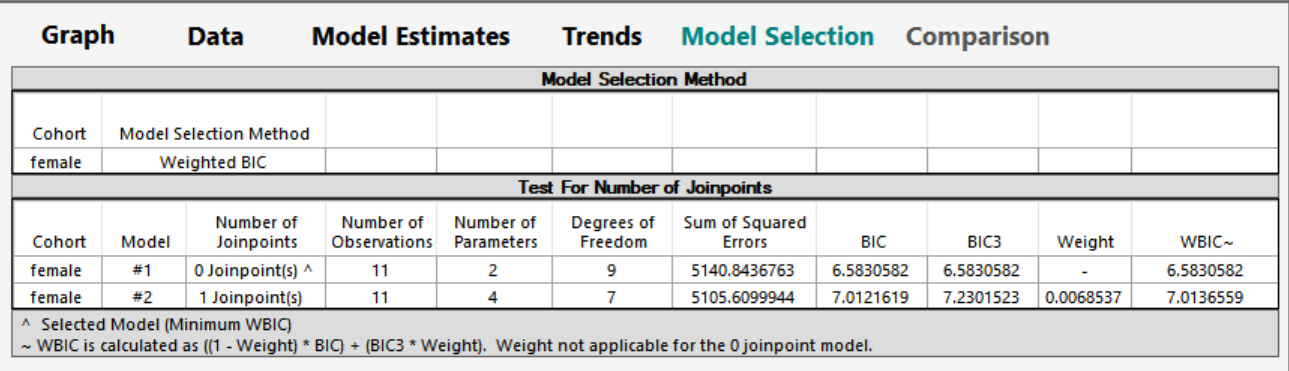


Figure S19. Trends in incidence rates among male aged 4 years old
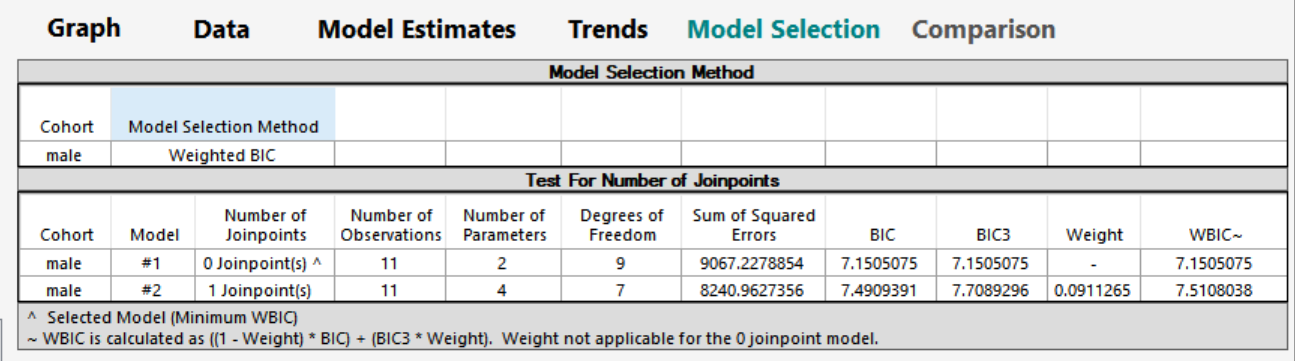


Figure S20. Trends in incidence rates among female aged 4 years old
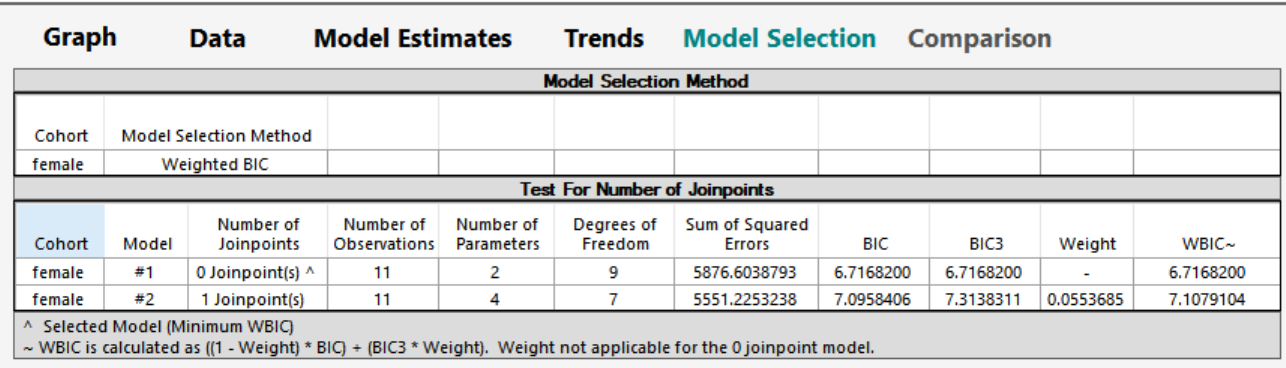


Figure S21. Trends in incidence rates among male aged 5 years old
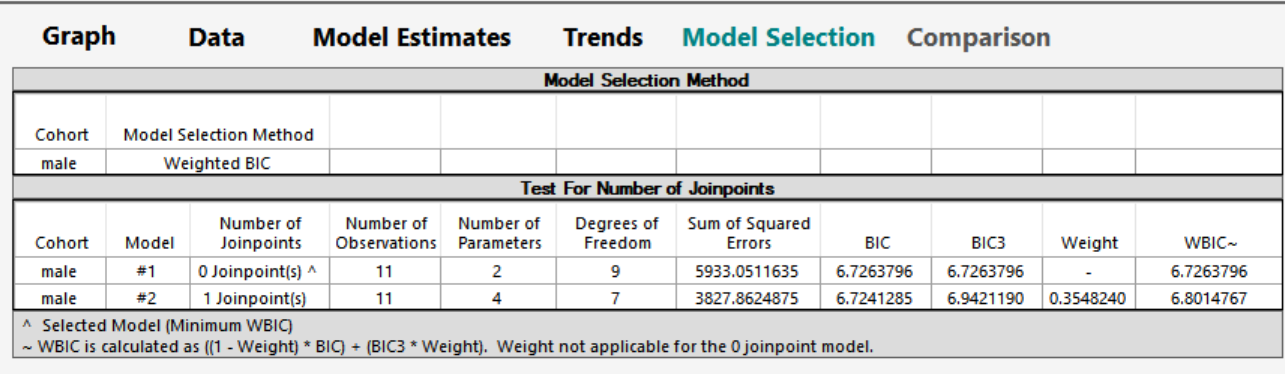


Figure S22. Trends in incidence rates among female aged 5 years old
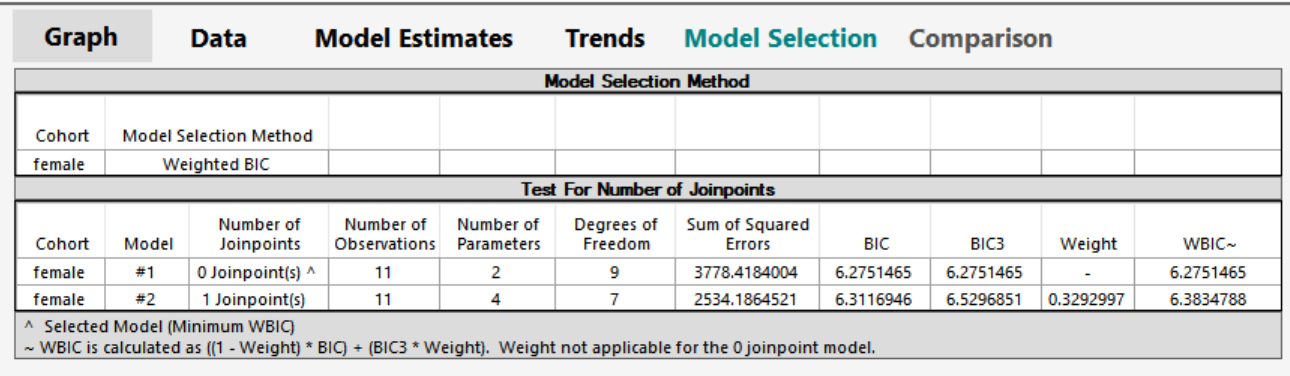


Figure S23. Trends in incidence rates among male aged 6 years old or older


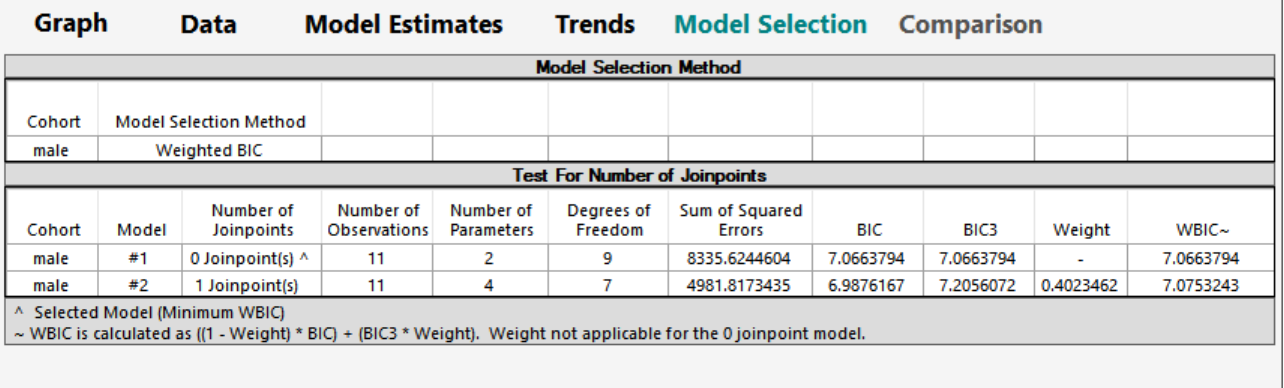


Figure S24. Trends in incidence rates among female aged 6 years old or older


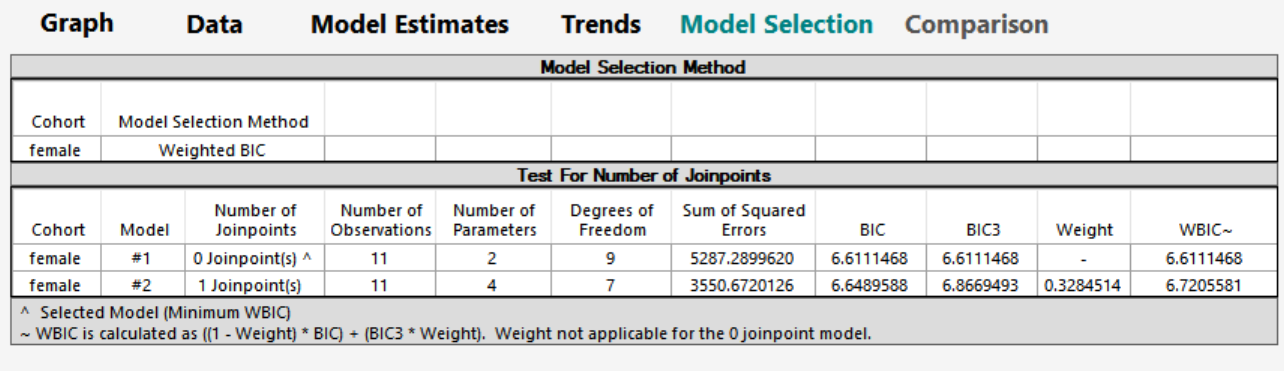

Supplement: S1 File — (DOCX) [file pone.0333544.s001.docx]
