# Supplementary material for: Analysis and forecast of hand, foot, and mouth disease epidemic trends in Guangzhou, China, 2013–2023
Source: PLoS One. 2025 Sep 30;20(9):e0333544. doi: 10.1371/journal.pone.0333544 (PMC12483251; doi:10.1371/journal.pone.0333544)
Supplement: S2 File — (DOCX) [file pone.0333544.s002.docx]

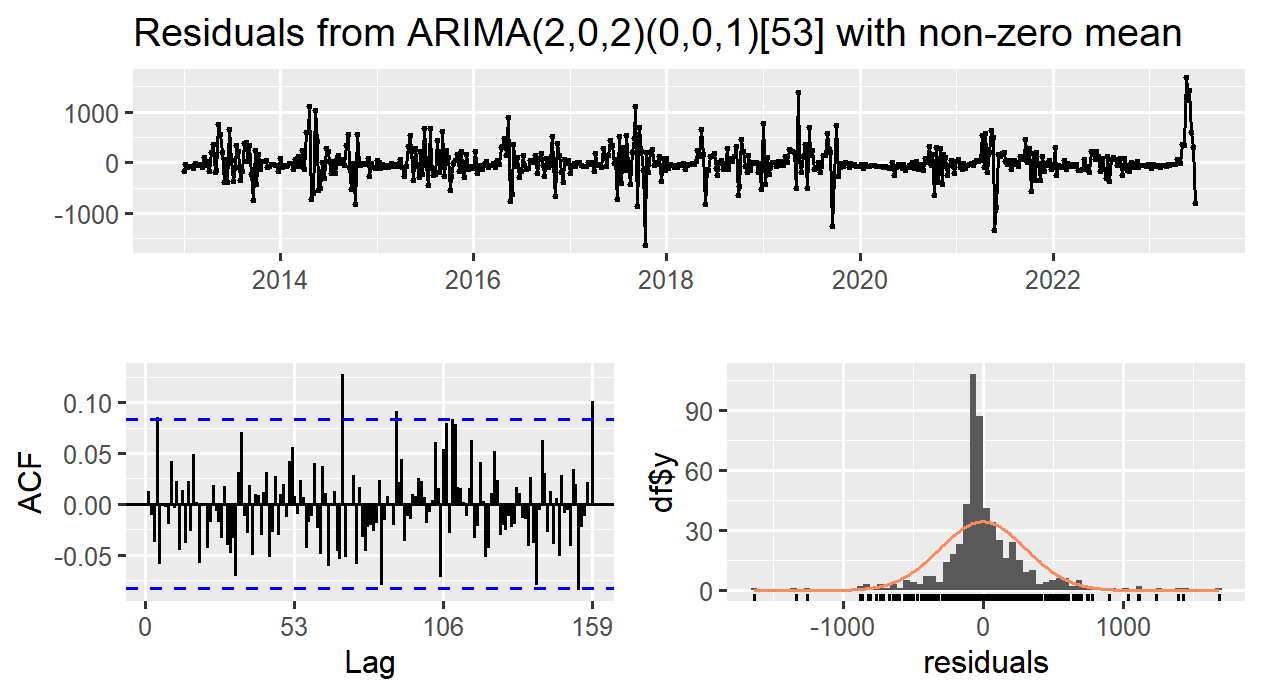


**Fig S1. Residual diagnostics plots and ACF following the differencing transformation**

The figure presents the diagnostic analysis of residuals from an SARIMA(2,0,2)(0,0,1)[53] model with a non - zero mean, encompassing three key components: the residual time - series plot (top), the autocorrelation function (ACF) plot (bottom left), and the residual distribution histogram with a kernel density curve (bottom right).


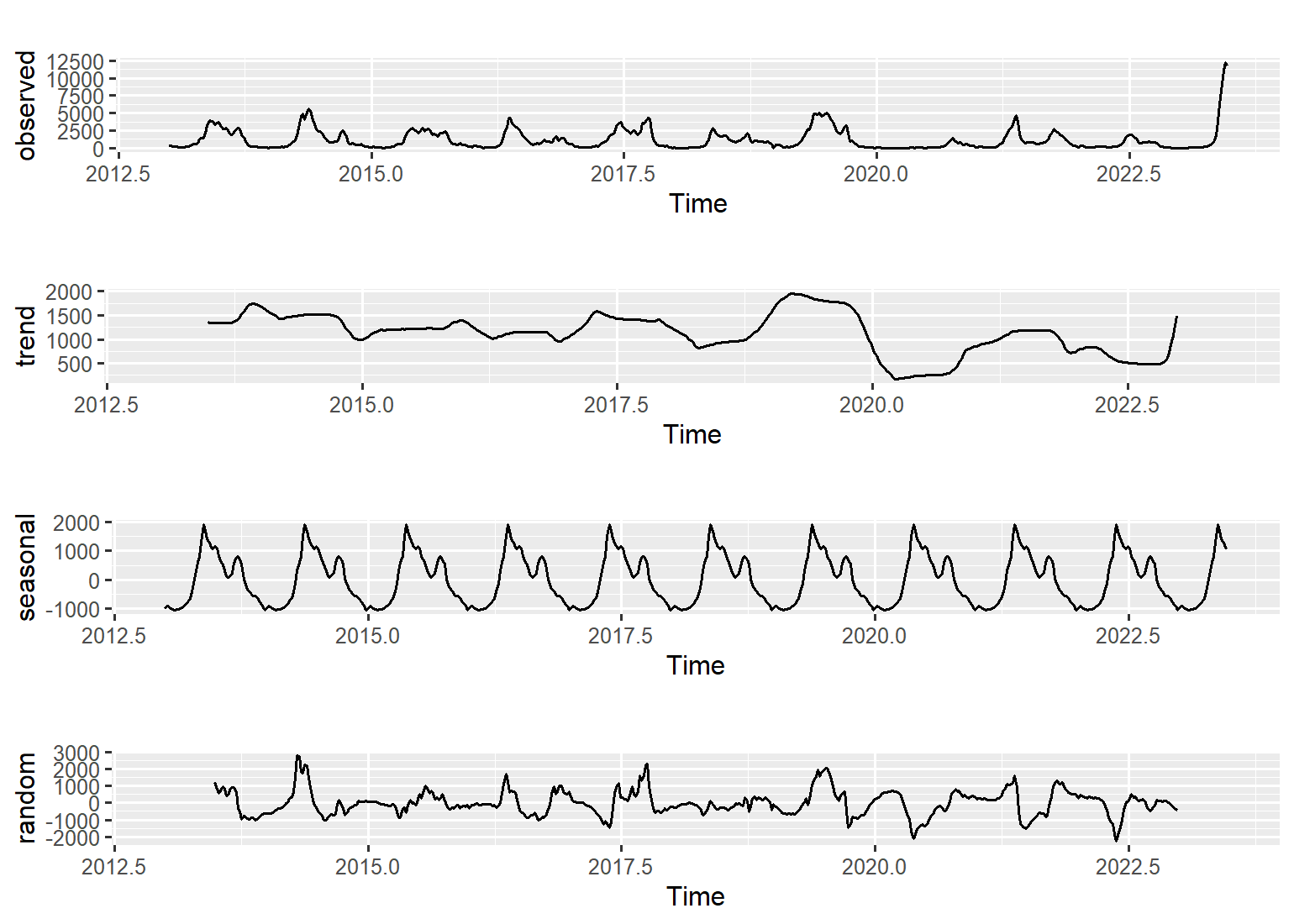


**Fig S2. Time Series Decomposition Plots**

This figure displays the time - series decomposition of disease counts from 2013 to 2023, featuring four panels: observed values (top), trend (second), seasonal fluctuations (third), and random residuals (bottom).

**Table S1. Overall trends ARIMA (2,0,2) (0,0,1) 53 Model parameter estimation**

|  |  | Estimate | Std. Error | *z* value | *P* value |
| --- | --- | --- | --- | --- | --- |
| AR | Lag2 | -0.78 | 0.08 | -9.54 | <0.001 |
| MA | Lag2 | -0.22 | 0.08 | -2.86 | 0.004 |
| MA, seasonality | Lag1 | 0.12 | 0.04 | 2.79 | 0.005 |
